# Potential Serum Biomarkers Associated with Premature Rupture of Fetal Membranes in the First Trimester

**DOI:** 10.3389/fphar.2022.915935

**Published:** 2022-07-08

**Authors:** Zhuoling An, Rui Zhao, Feifei Han, Yuan Sun, Yanping Liu, Lihong Liu

**Affiliations:** ^1^ Department of Pharmacy, Beijing Chao-Yang Hospital, Capital Medical University, Beijing, China; ^2^ Department of Clinical Nutrition, Peking Union Medical College Hospital, China Academic Medical Science and Peking Union Medical College, Beijing, China

**Keywords:** potential biomarkers, prediction model, premature rupture of fetal membranes, untargeted metabolomics, early pregnancy

## Abstract

Premature rupture of the fetal membranes (PROM) is a common and important obstetric complication with increased risk of adverse consequences for both mothers and fetuses. An accurate and timely method to predict the occurrence of PROM is needed for ensuring maternal and fetal safety. Untargeted metabolomics was applied to characterize metabolite profiles related to PROM in early pregnancy. 41 serum samples from pregnant women who developed PROM later in gestation and 106 from healthy pregnant women as a control group, were analyzed. Logistic regression analysis was adjusted to analyze a PROM prediction model in the first trimester. A WISH amniotic cell viability assay was applied to explore the underlying mechanisms involved in PROM, mediated by C8-dihydroceramide used to mimic a potential biomarker (Cer 40:0; O_2_). Compared with healthy controls, 13 serum metabolites were identified. The prediction model comprising four compounds (Cer 40:0; O2, sphingosine, isohexanal and PC O-38:4) had moderate accuracy to predict PROM events with the maximum area under the curve of a receiver operating characteristics curve of approximately 0.70. Of these four compounds, Cer 40:0; O2 with an 1.81-fold change between PROM and healthy control serum samples was defined as a potential biomarker and inhibited the viability of WISH cells. This study sheds light on predicting PROM in early pregnancy and on understanding the underlying mechanism of PROM.

**Trial Registration:** This study protocol has been registered at www.ClinicalTrials.gov, CT03651934, on 29 August 2018 (prior to recruitment).

## Introduction

Premature rupture of the fetal membranes (PROM), defined as rupture of the membranes without the onset of labor, is a common and important obstetric complication showing an association with increased risks of adverse consequences for both the mothers and fetuses including premature birth, maternal or neonatal infections, abruptio placenta, neonatal sepsis, maternal chorioamnionitis, endometritis and even fetal or neonatal death (Ha et al., 2018; American College of Obstetricians and Gynecologists’ Committee on Practice Bulletins—Obstetrics, 2016; Dars et al., 2014; Bopegamage et al., 2013; Yu et al., 2015). Facing these risks of severe complications, particularly sepsis, or in cases of severe oligohydramnios or chorioamnionitis, induction of labor immediately is indicated, (American College of Obstetricians and Gynecologists’ Committee on Practice Bulletins—Obstetrics, 2016; Goldenberg et al., 2008) Prevention of pregnancy complications through enhanced understanding and appropriate intervention is of critical importance to improve both maternal and neonatal outcomes (Feng et al., 2018).

At present, multifactorial causes of spontaneous PROM have been reported, including pathologic anatomic remodeling, (Makieva et al., 2017; Ha et al., 2018) complications from invasive procedures, (Gratacós et al., 2006) inflammation, (Romero et al., 2015) apoptosis, (Wang et al., 2016) oxidative stress (Ilhan et al., 2015; Zhang et al., 2020) and genetic factors (Tchirikov et al., 2018). However, the diagnosis and prevention of PROM remains a crucial clinical and challenging problem in obstetrics. The traditional diagnostic criteria are according to the patient’s complaint, the pH of vaginal fluids, observation of the presence of posterior vaginal fornix effusion, or obstetric ultrasonography (Vogel et al., 2018). Fluid might not be present in the vagina or can be contaminated with urine, cervical mucus, vaginal discharge, blood, semen or meconium, (Eleje et al., 2016) which can result in false diagnoses. In recent years, several researches on clinically diagnose PROM have been developed, some potential biomarkers such as placental protein 14 in amniotic fluid collected from pregnant women in their third trimester (Wang et al., 2018) and high-mobility group box-1 from amniotic fluid and cord blood (Menon and Taylor, 2019) were identified. However, these methods were invasive and may cause infection or abortion, or PROM diagnose in the third trimester before labor which cannot be used for PROM prediction in early pregnancy (Ghafoor, 2021). An ideal test should be non-invasive, expeditious, accurate, cost-effective, conveniently applicable and readily available. The low accuracy of diagnoses may delay treatment and induce further adverse outcomes for the pregnant woman and her fetus (Mandel et al., 2005). Faced with this clinical dilemma, an accurate and timely method to predict the occurrence of PROM is needed urgently to ensure the safety and health of women and their fetuses.

Given that PROM is a proximal cause of many severe maternal and fetal clinical outcomes, determining the difference between PROM and a healthy pregnancy will help in clarifying PROM’s underlying mechanisms and in developing effective diagnostic methods. This study was based on our previous research on untargeted metabolomics using high performance liquid chromatography–high resolution mass spectrometry (HPLC–HRMS) (Zhao et al., 2021). The altered serum metabolites in the first trimester involved in women diagnosed with PROM were screened compared with healthy pregnant women. Through a multivariate data model and cell viability experiments, we attempted to reveal the metabolic pathways and biomarkers related to PROM, and the mechanisms and role of potential biomarkers in the occurrence of this disease.

## Materials and Methods

### Study Design and Participants

A retrospective cohort study was conducted in pregnant women in early pregnancy (5–14 weeks of gestation) recruited at the Shunyi District Maternal and Child Health Hospital (Beijing, China) from October to December 2018. All enrolled subjects were ethnically Han Chinese and, at enrollment, were screened to be healthy, without chronic conditions or associated medication intake. All subjects provided written informed consent before inclusion and were requested to adhere to issued study restrictions. The samples used in this study were of materials remaining after routine physiological testing. The study was conducted in accordance with the Declaration of Helsinki, International Conference on Harmonization. The Ethics Committee of Peking Union Medical College Hospital approved the research protocol (ClinicalTrials.gov registry, number NCT03651934). Participants completed a baseline demographic and health questionnaire at enrollment. During the whole period of pregnancy until delivery, all the included participants had routine pregnancy examinations in the same hospital. The clinical outcomes of the participants were diagnosed and recorded by trained obstetricians.

**FIGURE 1:**
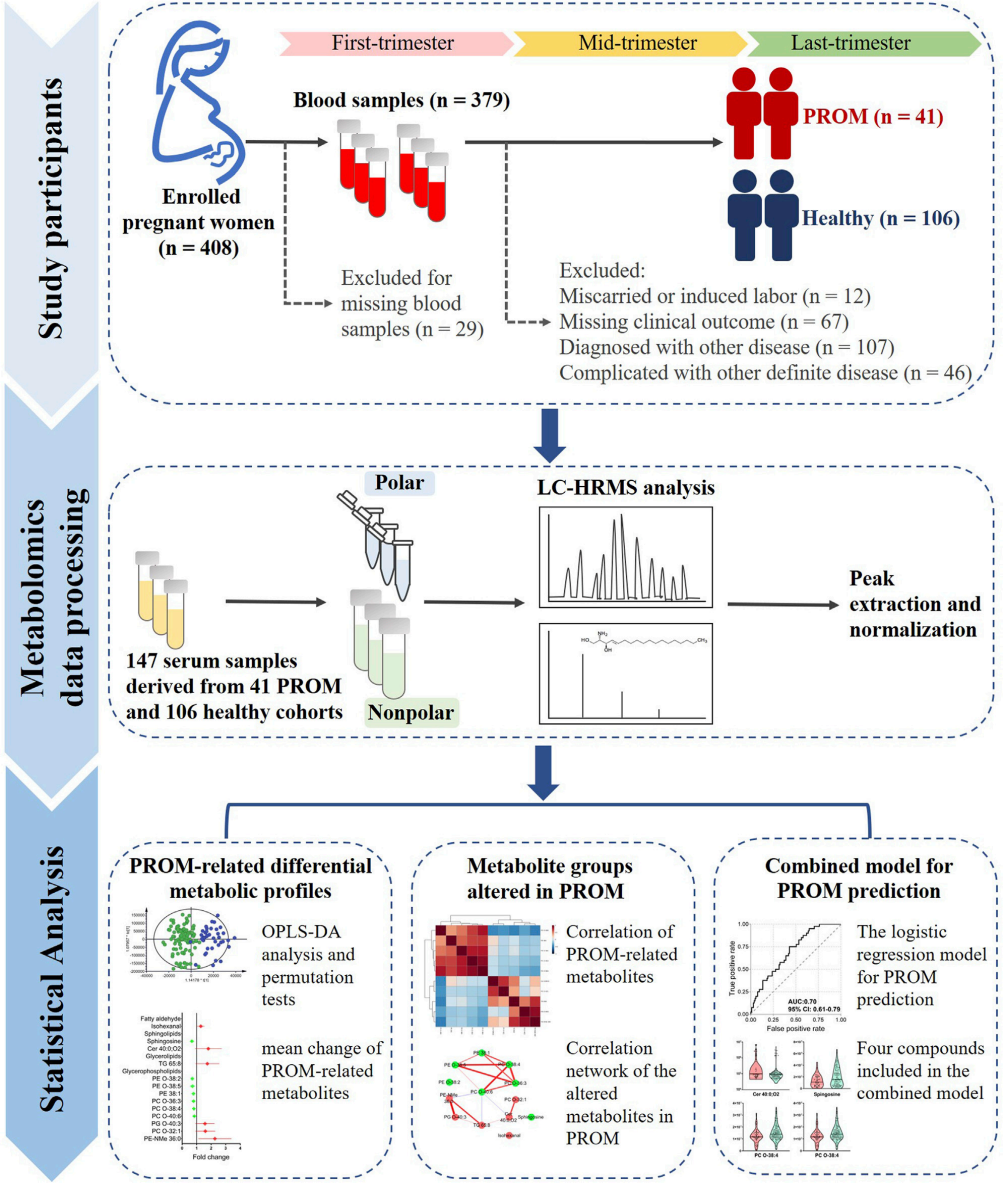
The study design of metabolic profiling of serum samples from women with PROM in the first trimester. There were three steps: 1) 408 pregnant women were enrolled, and 379 serum samples were collected in the first trimester. Of these women, 196 were excluded for miscarriage or induced labor (*n* = 12), missing clinical outcome data (*n* = 67), diagnosis of another disease (*n* = 107) or PROM complicated with other definite diseases (*n* = 46). Finally, 41 samples from women with PROM and 106 from healthy controls were included. 2) Metabolomics data processing using untargeted metabolomics was based on HPLC–HRMS, and the peaks were extracted and normalized; the features were identified according to compound databases. 3) The statistical analysis included identification of PROM-related differential metabolites and changes in metabolite groups in PROM in the first trimester; the final combined model included four potential biomarkers for predicting PROM.

Serum samples derived from healthy women and women with PROM were included. The following exclusion criteria were applied: 1) participants with miscarriages or induced labor; 2) participants lacking valid data about clinical outcomes; and 3) participants diagnosed with other diseases exclusive of PROM. The study design and cohort proportions are presented in Figure 1.

### Chemical Materials Used for Untargeted Metabolomics

MS-grade acetonitrile, methanol (MeOH), isopropanol and formic acid (HPLC-grade) were purchased from Merck (Darmstadt, Germany). The human amnioblast WISH cell lines (Cat. No. GNHu38) were acquired from the American Type Culture Collection (ATCC, Rockville, MA, United States). Standard C8-dihydroceramide was purchased from Toronto Research Chemicals (TRC, Toronto, ON, Canada). Dulbecco’s Modified Eagle’s Medium and fetal bovine serum was obtained from HyClone (Logan, UT, United States). All other standards used were of analytical grade or better.

### Metabolite Extraction and HPLC–HRMS Analysis

For 41 women with PROM and 106 healthy controls, 147 serum samples were prepared to organic (including non-polar metabolites) and aqueous phases (including polar metabolites). To analyze the metabolic extracts, an Agilent 1200 LC system coupled with a Q Orbitrap mass analyzer (Q Exactive, Thermo Fisher Scientific, Waltham, MA, United States) were performed in full MS scan mode (from *m/z* 67 to *m/z* 1,000) and in both positive and negative ionization modes. Chromatographic separation was performed using an ACQUITY HSS T3 C18 column (Waters; Milford, MA, United States, 2.1′ 100 mm, 1.8 mm). The experimental details, including sample preparation, LC-MS analysis, metabolic data processing and feature identification, were carried out according to our previous study (Zhao et al., 2021).

### Cell Viability Assay

Cell viability was determined by the Cell Counting Kit-8 assay. Human amnioblast WISH cells were seeded at a density of 1 × 10^4^ cells/well in 96-well plates and cultured overnight. WISH cells were then treated with a dihydroceramide analog, C8-dihydroceramide (C8-dhCer), (Wang et al., 2015; Muñoz-Guardiola et al., 2020) at 100, 50, 25, 12.5, or 6.25 μM for 24 h, and 0.1% DMSO was added as a vehicle to the control wells. Media were then refreshed, and 10 μl of Cell Counting Kit-8 solution was added per well. The optical densities at 450 nm wavelength were measured 4 h later using a microplate reader (BioTek, Winooski, VT, United States). Cell viabilities were expressed as a percentage of the controls cultured with 0.1% DMSO.

### Statistical Analysis

The orthogonal partial least squares discriminant analysis (OPLS-DA) was employed using SIMCA software 14.1 (Umetrics, Umeå, Sweden) for classification, and the models were validated by permutation tests (100 times) to evaluate the significance of the model (Eriksson et al., 2006). Variable importance in projection (VIP) scores were used to visualize the influence of a variable in the model. IBM SPSS Statistics (v. 21, IBM Corp., Armonk, NY, United States) was used to test differences between means by Student’s *t* tests and analyze a linear regression between intensity of two compounds. Differentially expressed metabolites were determined according to a VIP score > 1.0 and *p* < 0.05, and those with more than 1.5-fold change between PROM and healthy cohorts were defined as potential biomarkers. Pearson’s correlation coefficients (*r*) between the abundance of differential metabolites were examined and the correlation network was visualized using Cytoscape 3.7.1 (U.S. National Institute of General Medical Sciences) (Kohl et al., 2011). The dependent variable was PROM and the independent variables were measurements of 13 differential metabolite. Logistic regression was used to combine the predictive ability of candidate biomarkers, and receiver operating characteristic (ROC) curves and the area under the curve with 95% confidence intervals were calculated using IBM SPSS Statistics (Liang et al., 2020; Zhao et al., 2020).

## Results

### Baseline Characteristics of Participants

To determine potential biomarkers to predict the risk of PROM in early pregnancy, we established a normal pregnancy cohort at enrollment and a design according to clinical outcomes. In all, 147 serum samples were assigned to a PROM (*n* = 41) and to a healthy (*n* = 106) control cohort. The baseline characteristics in early pregnancy (Table 1) and maternal characteristics in the third trimester (Table 2) of the participants were obtained and evaluated. No significant differences were found between the healthy pregnant women and those with PROM for the following characteristics: maternal age, body mass index (BMI) at enrollment, gestational age, hemoglobin, C-reactive protein, fasting plasma glucose and homocysteine levels; and fasting plasma glucose, 1 and 2-h post-load plasma glucose (75-g oral glucose tolerance test, 75-g OGTT), white blood cell count, the hemoglobin levels and white blood cell count showed no significant differences in the two cohorts later in the third trimester.

**TABLE 1:** Baseline maternal characteristics in healthy control and PROM cohorts in the first trimester.

Characteristics	Healthy pregnant women (*n* = 106)	Pregnant women who developed PROM (*n* = 41)
Maternal age, years	28.7 ± 3.1	28.1 ± 3.2
BMI at enrollment, kg/m^2^	22.5 ± 3.6	21.9 ± 2.9
Gestational age, days	67.7 ± 13.6	69.7 ± 13.3
Hemoglobin, g/L	131.7 ± 9.4	132.1 ± 9.0
C-reactive protein, mg/L	3.0 ± 3.2	2.5 ± 2.3
Serum homocysteine, μM	8.8 ± 1.3	8.7 ± 1.5
White blood cell count, 10^9^	8.2 ± 1.9	8.4 ± 1.8
Fasting plasma glucose, mmol/L, mmol/L	4.4 ± 0.4	4.4 ± 0.4
Family history of diabetes, n (%)		
Yes	12 (11.3)	2 (4.9)
No	92 (86.8)	38 (92.7)
Missing	2 (1.9)	1 (2.4)
History of dyslipidemia, n (%)		
Yes	5 (4.7)	0
No	100 (94.3)	40 (97.6)
Missing	1 (0.1)	1 (2.4)
History of polycystic ovarian syndrome, n (%)		
Yes	3 (2.8)	0
No	103 (97.2)	39 (95.1)
Missing	0	2 (4.9)
Alcohol intake, n (%)		
Yes	3 (2.8)	0
Times per week	1 ± 0	0
No	103 (97.2)	41 (100.0)
Missing	0	0

OTGG, oral glucose tolerance test; BMI, body mass index.

Values are means ± SD or numbers (percentages).

**TABLE 2:** Maternal characteristics in healthy and PROM cohorts in the third trimester.

Characteristics	Healthy pregnant women (*n* = 106)	Pregnant women with PROM (*n* = 41)
Hemoglobin, g/L	129.0 ± 9.6	127.5 ± 8.9
White blood cell count, 10^9^	8.8 ± 2.3	8.6 ± 2.4
Fasting plasma glucose, mmol/L	4.2 ± 0.8	4.4 ± 0.3
1-h post-load plasma glucose (75-g OGTT), mmol/L	7.0 ± 1.3	7.2 ± 1.0
2-h post-load plasma glucose (75-g OGTT), mmol/L	6.4 ± 0.9	6.2 ± 1.3
Fetus presentation, n (%)		
Cephalic presentation	72 (67.9)	26 (63.4)
Breech presentation	2 (1.9)	0
Stillborn	1 (0.9)	1 (2.4)
Missing	31 (29.2)	14 (34.1)

OTGG, oral glucose tolerance test.

Values are means ± SD or numbers (percentages).

### Premature Rupture of the Fetal Membranes-Related Differential Metabolic Profiles in Early Pregnancy

**FIGURE 2:**
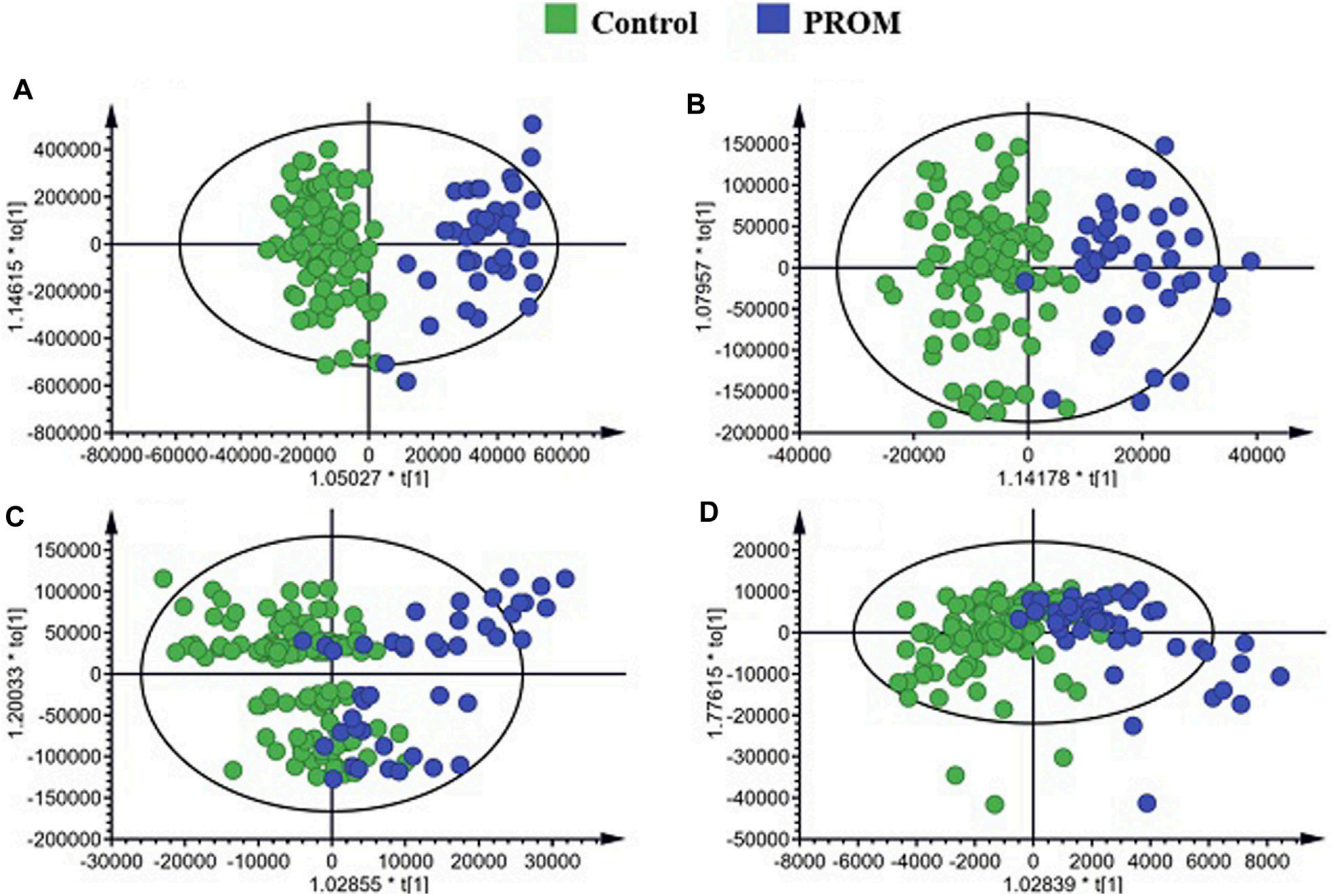
Orthogonal partial least squares discriminant analysis (OPLS-DA) score plots and random permutation tests of the non-polar metabolites. **(A)** Score plots of polar metabolites for positive ion mode; **(B)** polar metabolites for negative ion mode; **(C)** non-polar metabolites for positive ion mode; **(D)** non-polar metabolites for negative ion mode.

A total of 147 serum samples from 106 healthy pregnant women and 41 pregnant participants diagnosed with PROM were used for studying the metabolomic profiles of PROM. OPLS-DA was applied to screen for differential metabolic features between the two cohorts. As shown in Figure 2, the OPLS-DA score plots of polar and nonpolar metabolites for positive/negative ion mode analysis derived from the serum samples showed good separation between the pregnant women diagnosed with PROM and the healthy control participants.

**FIGURE 3:**
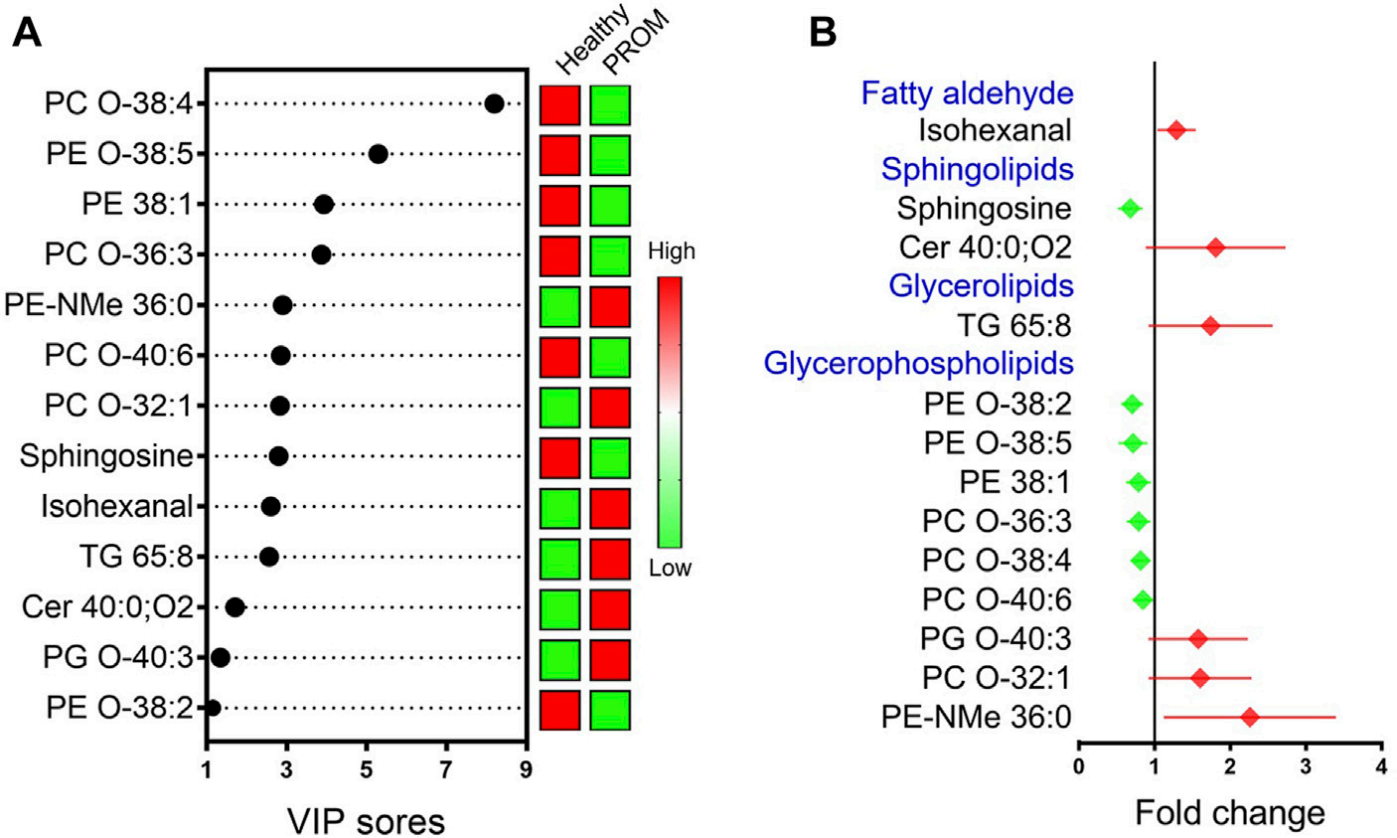
VIP values **(A)** and mean change (means with 95% confidence intervals) **(B)** in differential metabolic measures with VIP >1 and *p* < 0.05 in pregnant women diagnosed with PROM compared with healthy pregnant women. The red symbols indicate that the fold change of the particular metabolite in the PROM cohort were upregulated (fold change >1); green symbols (fold change <1) represent downregulated metabolites.

Following the analysis of the multivariate data model and identification by comparing the public spectral databases, 13 serum metabolites with significant difference (VIP > 1.0 and *p* < 0.05) between two groups are presented in Figure 3, and the details of the differential metabolites are summarized in Table 3. Compared with healthy pregnant women, the differentially regulated metabolites in pregnant women diagnosed with PROM are presented as fold change (FC > 1 indicates upregulated metabolites; FC < 1 indicates downregulated metabolites). The results indicated that the levels of isohexanal, a ceramide (Cer 40:0; O2), a glycerolipid (TG 65:8) and three glycerophospholipids were upregulated, whereas sphingosine and six glycerophospholipids were downregulated in the PROM cohort. Interestingly, we found that all these 13 differential metabolites related to PROM were lipids, suggesting that disordered lipid metabolism was associated with PROM.

**TABLE 3:** Differential metabolites in pregnant women with PROM compared with healthy pregnant women.

Categories	*m/z*	rt (min)	Name	Formula	Adduct ion	*p* value	Mean change
Fatty aldehyde	83.0859	3.60	Isohexanal	C6H12O	M-H2O + H [1+]	7.1E^−03^	1.29
Sphingolipids	282.2783	23.73	Sphingosine	C18H37NO2	M-H2O + H [1+]	2.7E^−02^	0.68
	624.6274	17.74	Cer 40:0; O2	C40H81NO3	[M + H]+	3.8E^−02^	1.81
Glycerolipids	1027.8665	15.78	TG 65:8	C68H116O6	[M−H]−	2.2E^−02^	1.74
Glycerophospholipids	756.5922	17.48	PE O-38:2	C43H84NO7P	[M−H]−	9.3E^−03^	0.71
	752.5576	15.86	PE O-38:5	C43H78NO7P	[M + H]+	1.6E^−02^	0.72
	774.6006	15.43	PE 38:1	C43H84NO8P	[M + H]+	4.9E^−02^	0.79
	770.6034	15.76	PC O-36:3	C44H84NO7P	[M + H]+	2.8E^−02^	0.79
	796.6186	15.84	PC O-38:4	C46H86NO7P	[M + H]+	1.5E^−02^	0.81
	820.619	15.39	PC O-40:6	C48H86NO7P	[M + H]+	4.0E^−02^	0.85
	813.6002	10.76	PG O-40:3	C46H87O9P	[M−H]−	4.2E^−02^	1.58
	718.5742	14.77	PC O-32:1	C40H80NO7P	[M + H]+	1.7E^−02^	1.60
	760.5865	10.9	PE-NMe 36:0	C42H84NO8P	[M−H]−	6.0E^−03^	2.26

### Metabolite Groups Altered in Premature Rupture of the Fetal Membranes in Early Pregnancy

**FIGURE 4:**
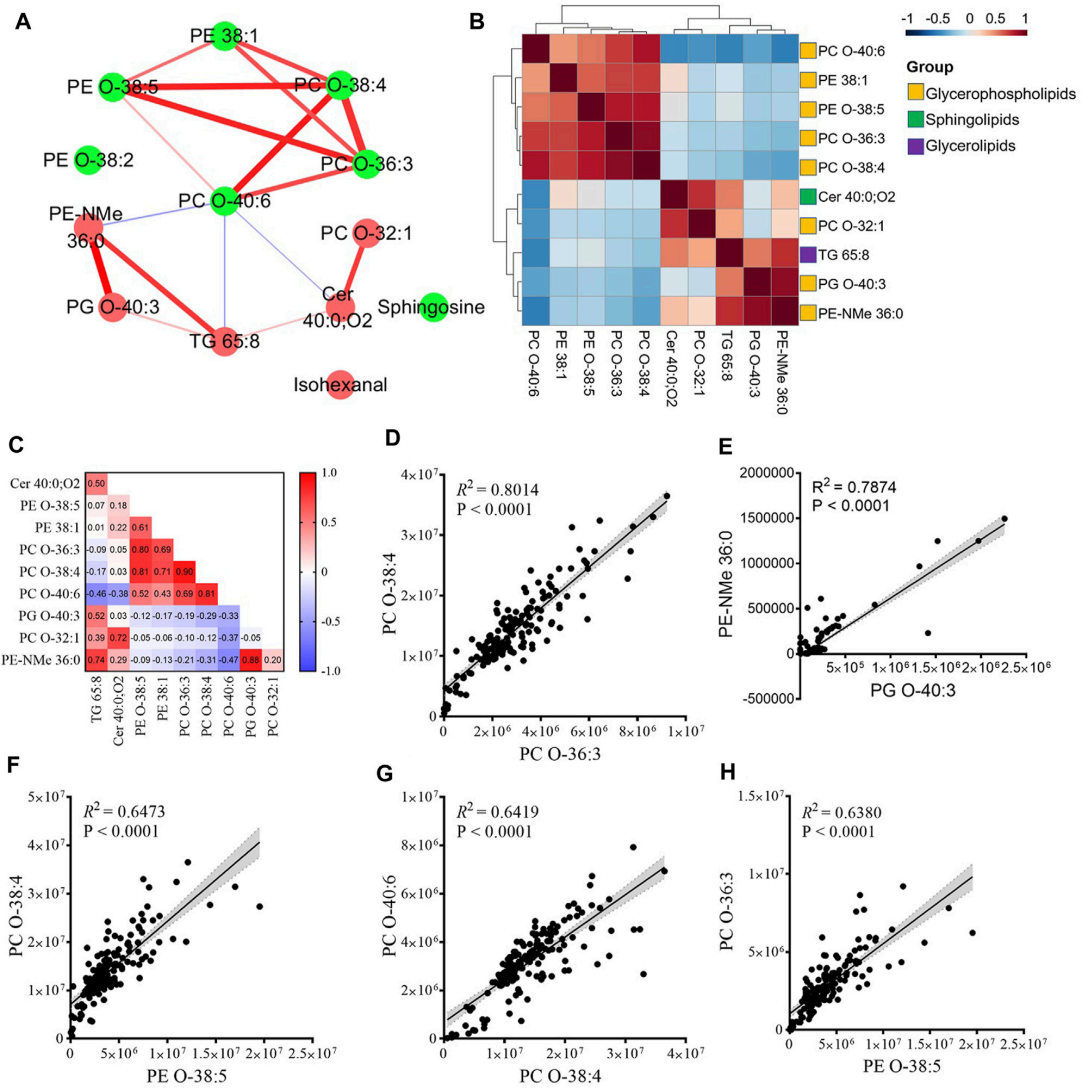
Functional metabolite groups altered in women with PROM in early pregnancy. **(A)** The correlation network of the altered metabolites in PROM. The node represents a compound, and each edge represents the strength of correlation between two compounds. Edge weights represent Pearson’s correlation coefficients. The red/green nodes represent upregulated/downregulated metabolites in the PROM cohort. The red/blue edges indicate positive/negative correlations between two metabolites. **(B,C)** Correlation matrix colored by the Pearson correlation coefficient (*r*) of each pair of PROM-related compounds across samples. **(D–H)** The linear model of top five highly correlated (*R* ^2^ > 0.6) pair of PROM-related metabolites. The 95% confidence interval for the linear regression is represented by the gray area.

To detect the functional groups of metabolites that changed during the first trimester, we performed correlation analysis on the temporal intensity profiles of the 13 PROM-related metabolites. These metabolites were significantly increased or decreased and tended to cluster together. On the basis of the correlation relationship indicated by the Pearson correlation coefficient (*r*) of each pair of PROM-related compounds across samples (Figure 4C), we constructed a correlation network to explore the potential regulatory relationships (Figure 4A). Visualization of the network indicated that there might be different steps in the same pathway between inter-and intra-metabolite groups with the glycerophospholipids. These findings highlighted that a highly coordinated metabolite regulatory network may underly the pathology of PROM.

We next examined the main clusters present in the correlation analysis (Figure 4B). The hierarchical clustering among four glycerophospholipid metabolites—PC O-38:4, PC O-36:3, PE-O 38:5, and PE 38:1—and all of these decreased differentially in the PROM cohort in early pregnancy. Five of six downregulated glycerophospholipid metabolites intra-correlated positively, and three of the upregulated metabolites correlated with PC O-40:6 (Figure 4A). This result suggests that different glycerophospholipids might closely relate to altered metabolomic dynamics in patients with PROM. The largest cluster comprised glycerophosphocholines (PCs) and glycerophosphoethanolamines (PEs), two classes of glycerophospholipids. The linear model of the top five most highly correlated (coefficient of determination, *R* ^2^ > 0.6) pair of PROM-related metabolites are shown in Figures 4D–H.

### Prediction for Premature Rupture of the Fetal Membranes in Early Pregnancy Serum Samples

**FIGURE 5:**
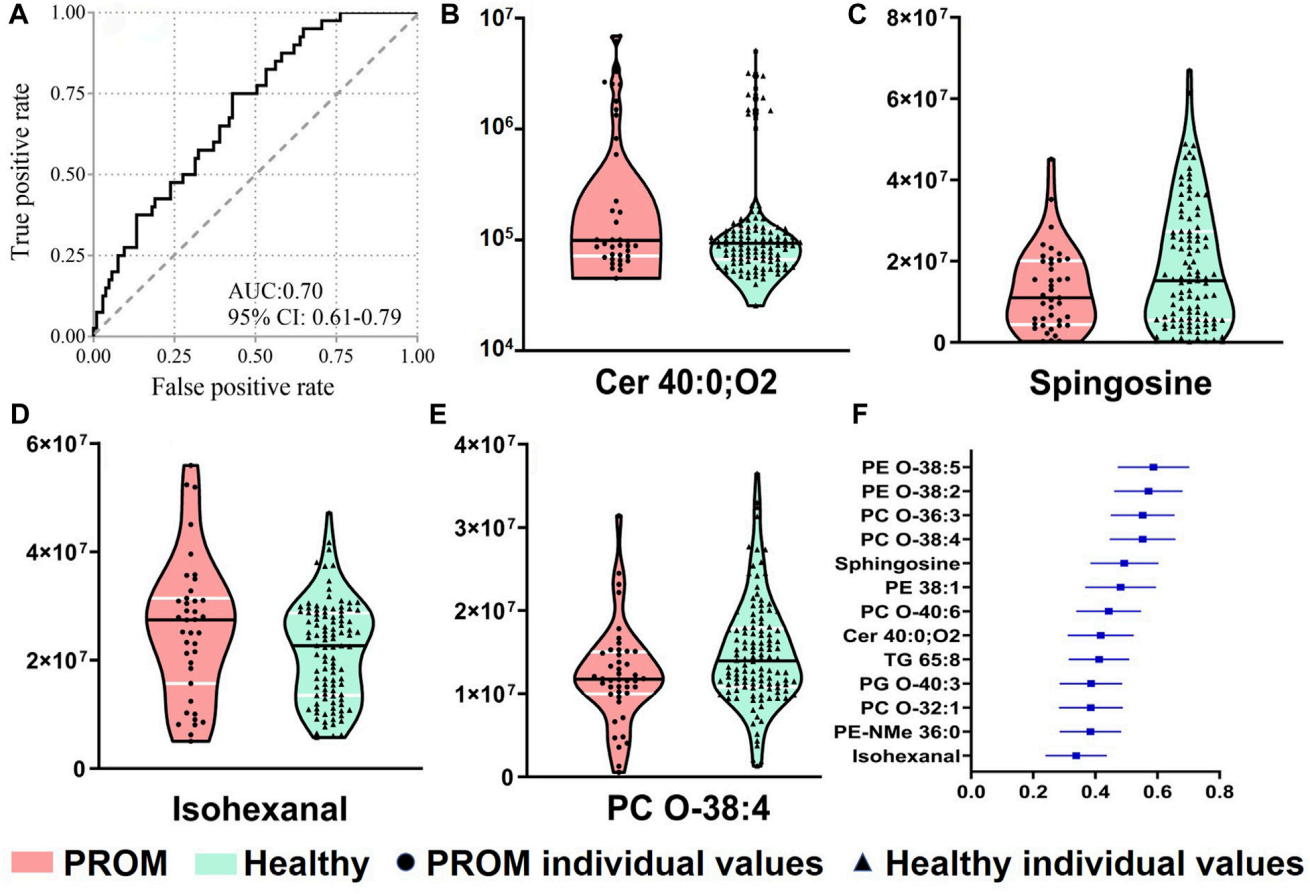
The logistic regression model based on four metabolites for predicting PROM in early pregnancy serum samples. **(A)** The ROC curve of the combination of four compounds in serum. Violin plots (medians with quartiles) are shown of four compounds, Cer 40:0; O_2_ **(B)**, sphingosine **(C)**, isohexanal (a fatty aldehyde) **(D)** and PC O-38:4 (a glycerophospholipid) **(E)**, with significant differences between the PROM and healthy cohorts. The forest plot of the AUC values of differential compounds **(F)**.

To identify the predictors for PROM in early pregnancy serum samples, the differential metabolites were included into the logistic regression model with further adjustment, and the combined panel was further analyzed using binary logistic regression. With four metabolites, the model had moderate accuracy to predict PROM with a maximum area under the ROC curve (AUROC) of ∼0.70 (Figure 5A) with 75.0% sensitivity and 57.1% specificity. The four metabolite markers were isohexanal, sphingosine, Cer 40:0; O_2_ and PC O-38:4. The violin plots of them (Figures 5B–E) showed significant differences between two cohorts, and the forest plot of the AUC values of differential compounds was shown in Figure 5F. These results suggest that it might be clinically possible to use small amounts (50 μl minimum) of maternal serum to predict the PROM risk in early pregnancy.

### The Cell Viability Was Reduced by C8-Dihydroceramide

**FIGURE 6:**
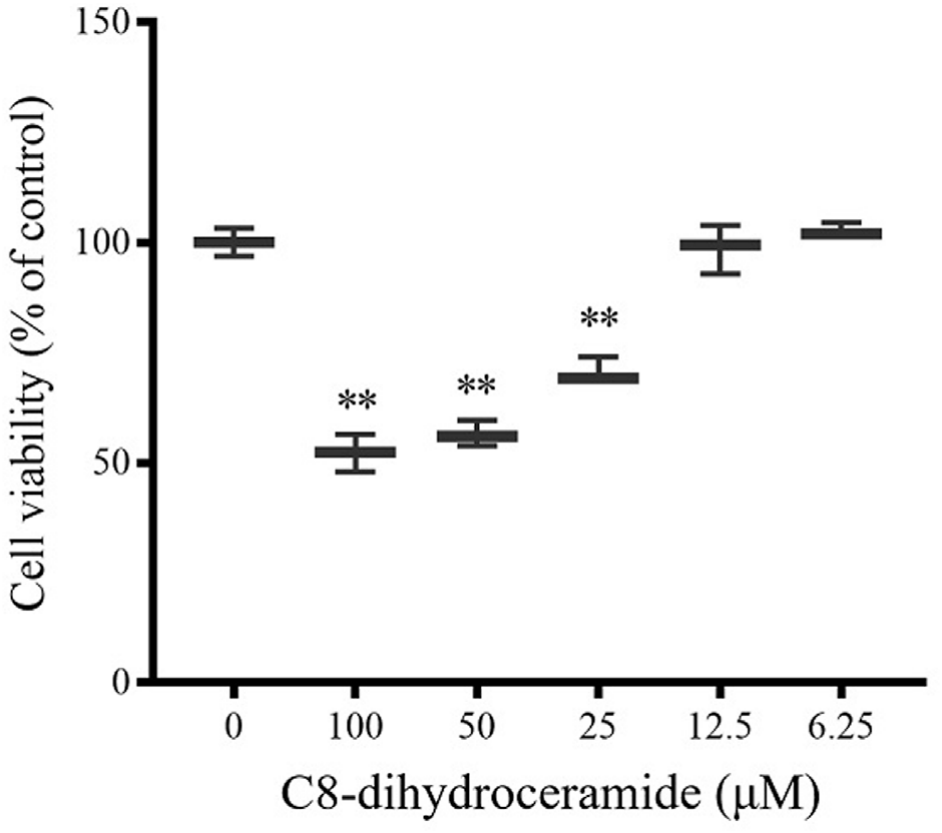
The viability of WISH cells was inhibited by C8-dihydroceramide. WISH cell lines seeded on 96-well plates were treated with C8-dihydroceramide (0, 6.25, 12.5, 25, 50, or 100 μM) for 24 h. Bars represent the mean values with min to max intervals. **p < 0.01.

Of the four compounds that comprised our PROM prediction model, Cer 40:0; O2 showed the most significant change in the PROM cohort with an 1.81-fold change compared with the healthy controls. As a potential biomarker (>1.5-fold change), the effect of Cer 40:0; O2 on cell viability was further examined in the human amnioblast WISH cell line. As a naturally occurring very long-chain ceramide, Cer 40:0; O2 has experimental limitations resulting from poor cell permeability (Petrache et al., 2005); short-chain dihydroceramide (C8-dihydroceramide) was used for this *in vitro* assay. Treatments with 100, 50, and 25 μM of C8-dihydroceramide significantly reduced the viability of WISH cells to 52.2% (range 47.9%–56.4%), 56.5% (range 53.5%–59.5%) and 70.6% (range 68.8%–74.0%), respectively (Figure 6).

## Discussion

### Main Findings

The principal findings of this study included following: 1) Compared with healthy controls, thirteen differential serum metabolites were identified in PROM women in the early pregnancy; 2) The PROM prediction model comprising four compounds (Cer 40:0; O2, sphingosine, isohexanal and PC O-38:4) had moderate accuracy to predict PROM events with AUROC of ∼0.70; 3) As a potential biomarker, Cer 40:0; O2 showed inhibition of WISH cell viability, which suggests that cell survival or proliferation may be a potential mechanism of PROM mediated by dihydroceramide.

Collectively, these observations suggest that changes in the sphingolipid-related pathway might be closely associated with PROM. This study sheds light on predicting PROM in early pregnancy and on understanding the underlying mechanism of PROM.

### Interpretation

The etiology of PROM is complex and is considered to be related to membrane structure, inflammation, apoptosis, oxidative stress and other factors (Quist-Nelson et al., 2018). Glycerophospholipids are a major structural component of cell membranes and are crucial for many biological processes (Huang and Freter, 2015; Hu and Zhang, 2018). The metabolism of glycerophospholipids is very complex and is regulated by different signaling pathways, during which many forms of bioactive lipid molecules are generated, such as arachidonic acid, phosphatidic acid and lysophosphatidic acid (Oude Weernink et al., 2007). However, there is no direct evidence on the association between glycerophospholipids and PROM. Our study on correlations between potential biomarkers showed that the largest cluster was composed of PCs and PEs. PCs are among the most abundant phospholipids in the mammalian cell membrane, and the composition of membrane lipids is correlated with the pathophysiology of metabolic disorders (Stace and Ktistakis, 2006; Merrill, 2011). Considering the correlation does not imply causation, this suggests a possibility that glycerophospholipids might be closely related to the altered metabolome dynamics seen during early pregnancy associated with PROM.

Accumulating evidence indicates a close bidirectional homeostatic crosstalk between sphingolipids and glycerophospholipids, and dysregulation in this crosstalk contributes to lipotoxicity-induced metabolic stress (Köberlin et al., 2015; Vonkova et al., 2015; Rodriguez-Cuenca et al., 2017). Sphingosine and Cer 40:0; O2 have a diverse range of functions linked to cell survival, membrane integrity and metabolic regulation (Siddique et al., 2015).

To understand the biological significance of possible biomarkers in PROM, appropriate human amnioblast WISH cell models and Cer 40:0; O2 (with the most significant differences among the predicted models) were selected to test. Under some pathological conditions, intracellular ceramide is synthesized primarily enzymatically through a *de novo* pathway and the ceramides accumulate (Summers et al., 2019). Because of the experimental limitations on poor cell permeability of Cer 40:0; O2, its short-chain analog (C8-dihydroceramide) was used to examine the effects on the viability of human amnioblast WISH cells (Petrache et al., 2005). Cell viability was significantly reduced after 24 h when treated with C8-dihydroceramide at 25 μM or above. The inhibition of cell viability by C8-dihydroceramide suggests that cell survival or proliferation may be a potential mechanism of PROM mediated by dihydroceramide, consistent with the functions of dihydroceramides reported previously (Kumar et al., 2017; Nagahashi et al., 2018). Sphingolipids can induce lipotoxicity (Choi et al., 2018; Walchuk et al., 2020) and inflammation (Zhang et al., 2018; Anderson et al., 2020). Besides, a previous study described the roles of sphingolipid-related pathway in biological function including cell migration, apoptosis, autophagy (Li et al., 2020) as well as in human disorders such as cancer and cardiovascular disorders (Taniguchi and Okazaki, 2014). The levels of two sphingolipids exhibited a heterogeneous association pattern with PROM: sphingosine decreased and Cer 40:0; O2 increased in women with PROM compared with those in healthy pregnant controls. Sphingosine-1-phosphate (S1P) plays an important role in cell proliferation, apoptosis, angiogenesis and immune cell migration. S1P can protect germ cells, inhibit cell apoptosis, reduce early embryo debris and improve maternal and fetal immune tolerance during pregnancy. As the substrate of S1P, sphingosine is decreased in early pregnancy, and this might result in the reduction of S1P and further induce PROM in later gestation. The biological significance of these metabolites implies an association between sphingolipid-related pathways and PROM and the potential to predict PROM in early pregnancy. These results suggested the novel concept that PROM might be associated with apoptosis and that this might be a therapeutic target.

Our study also indicated that isohexanal increased significantly in PROM in early pregnancy. PROM is associated with oxidative stress, (Vogel et al., 2018) during which unstable peroxidative intermediates are decomposed into active aldehydes, including isohexanal. In the adrenal cortex, the activation of steroidogenesis results in the generation of large amounts of lipid aldehydes, including isohexanal (Pastel et al., 2016). A previous study showed that laboratory rats release isohexanal and hexanal when they are stressed and evoke a variety of anxiety-related responses (Inagaki et al., 2014). Isohexanal could be a main component of the alarm pheromone, which might shed light on the mechanism of stress-related PROM mediated by isohexanal. We hypothesize that the above information might help us to understand the underlying mechanism of PROM in early pregnancy.

### Clinical Implications

In this study, we systematically analyzed differential metabolites associated with PROM according to untargeted metabolomics. Thirteen lipids exhibited significant differences between the PROM and control cohorts in the first trimester. We further developed a combined model comprising four metabolites to predict the risk of PROM in early pregnancy using small serum samples (50 μl minimum). Interestingly, using this combined model alone without input from any other clinical features, we could predict the risk of PROM in early pregnancy.

### Strengths and Limitations

The main strength of this study is the retrospective cohort included pregnant women who were screened to be healthy, and serum samples from those who developed PROM later in gestation and healthy pregnant women were compared. The participants diagnosed with other diseases exclusive of PROM were excluded, which might minimum the effects on serum metabolism caused by confounding factors from other diseases. The second important strength is the differential serum metabolites associated with PROM were identified and a prediction model for PROM risk in the first trimester was analyzed.

This study also has some limitations. First, this research was based on a small cohort and small variations in clinical characteristics, further validation in a larger independent cohort with diverse ethnicities and longitudinal study is required. Such studies could help explain the mechanism of PROM in early pregnancy. Second, the metabolome of serum changes in all stages of pregnancy were not monitored, but only in the first trimester. Therefore, the dynamically changes of differential metabolites through pregnancy should be investigated to explore the underlying mechanism in PROM.

## Conclusion

Thirteen serum differential metabolites were identified in the women with PROM. Our further found prediction model comprising four compounds (Cer 40:0; O2, sphingosine, isohexanal and PC O-38:4) might help predict PROM events in early pregnancy. The results suggested that a sphingolipid-related pathway might be closely associated with PROM, and that the levels of four metabolites might be valid PROM predictors. This study has shed light on the prediction of PROM in early pregnancy and on its underlying mechanisms.

## Data Availability

The raw data supporting the conclusion of this article will be made available by the authors, without undue reservation.
